# Interface Pressures Derived from a Tubular Elastic Bandage

**DOI:** 10.3400/avd.oa.20-00136

**Published:** 2020-12-25

**Authors:** Kotaro Suehiro, Noriyasu Morikage, Takasuke Harada, Makoto Samura, Takashi Nagase, Yuriko Takeuchi, Takahiro Mizoguchi, Ryo Suzuki, Hiroshi Kurazumi, Kimikazu Hamano

**Affiliations:** 1 Division of Vascular Surgery, Department of Surgery and Clinical Science, Yamaguchi University Graduate School of Medicine, Ube, Yamaguchi, Japan

**Keywords:** compression therapy, tubular bandage, interface pressure

## Abstract

**Objective:** We sought to clarify the interface pressure (IP) when using a tubular elastic bandage (TEB) and examine the possibility for TEBs to provide IPs comparable to those provided by anti-thrombotic stockings.

**Materials and Methods:** In 40 healthy patients, IPs were measured at the level of calf at its maximum diameter (C) and transition of the medial gastrocnemius muscle into the Achilles tendon (B1) while a single or double layer of TEBs (17.5 cm in circumference) were applied with the patient in a supine position.

**Results:** Including both the C and B1 levels, circumferences and IPs showed a good correlation (single layer; r=0.72, double layer; r=0.75). The IP obtained with a single layer of TEB at the C level (median, 17 mmHg [range, 12–23 mmHg]) was higher than that at the B1 level (14 mmHg [11–18 mmHg], p<0.001). When double-layer TEB was used, the IP at B1 level increased to 18 (14–23) mmHg (p<0.001 vs. single layer).

**Conclusion:** Considering the characteristics of TEBs and using a single or double layer appropriately, creating a pressure profile mimicking that of an anti-thrombotic stocking seemed to be feasible when using a TEB.

## Introduction

At the same time as some controversies existing, with anti-thrombotic stockings (ATS) still being considered to reduce the risk of venous thromboembolism in postoperative patients,^1–4)^ ATS are known to cause skin irritation and/or uncomfortable feeling, and, more seriously, can damage soft tissue and/or peripheral nerves. These complications are considered to be caused mainly by improper fitting and/or application technique.^5)^ However, this may not be simply because of the lack of experience of nurses and/or patients themselves. Postoperative leg sizes in the patients who have undergone vascular or orthopedic surgery can vary greatly depending on the severity of postoperative inflammation and/or edema. Bandages can fit legs of any size and shape, but maintaining a proper interface pressure (IP) is difficult, with the guideline recommending daily re-measurement of leg sizes and refitting of ATS whenever necessary,^4)^ however, this would lead to excessive economic costs and work overload for staff. For these reasons, the nurses at our institute started to use tubular elastic bandages (TEBs) instead of ATS. Indeed, TEBs are less costly and easier to apply than ATS. Moreover, their thick fabric is beneficial to avoid medical device-related pressure ulcer. However, they are not designed to generate graduated compression to increase venous blood flow as demonstrated by Sigel et al.^6)^ and Lawrence et al.,^7)^ i.e., 18 mmHg at the ankle and 14 mmHg at the calf. Since the IPs obtained TEBs have not been studied well, we investigated IPs obtained when using TEBs and discussed whether TEBs could provide IPs mimicking those provided by ATS.

## Materials and Methods

This prospective study was approved by the Institutional Review Board of Yamaguchi University Hospital (Center for Clinical Research, Ube, Yamaguchi, Japan; H2020-040). All patients provided signed, informed consent before enrollment. The TEB evaluated in this study was an Elutube® (NIPPON SIGMAX Co., Ltd., Tokyo, Japan), which consists of 84% cotton, 12% polyester, and 4% polyurethane, because Elutube® has already been adopted and utilized in our institute. In this study, size E, which fits medium-sized calves (not defined by measurements) was used. The circumference of the TEB in its original shape is 17.5 cm. The study patients comprised 40 healthy volunteers with a median age of 38 (range, 23–60) years. The characteristics of the patients and their right legs are summarized in **Table 1**.

**Table 1:** Table 1 Patient and leg characteristics (N=40)

Subjects	
Age (years; median [range])	38 (23–60)
Sex (male : female)	20 : 20
Height (cm; median [range])	165 (147–181)
Body mass index (kg/m^2^; median [range])	21 (18–33)
Leg circumference	
Calf (cm; median [range])	36.2 (30.2–46.8)
B1 (cm; median [range])	25.7 (22.3–31.3)
Ankle (cm; median [range])	21.0 (18.2–24.8)

**Figure 1:**
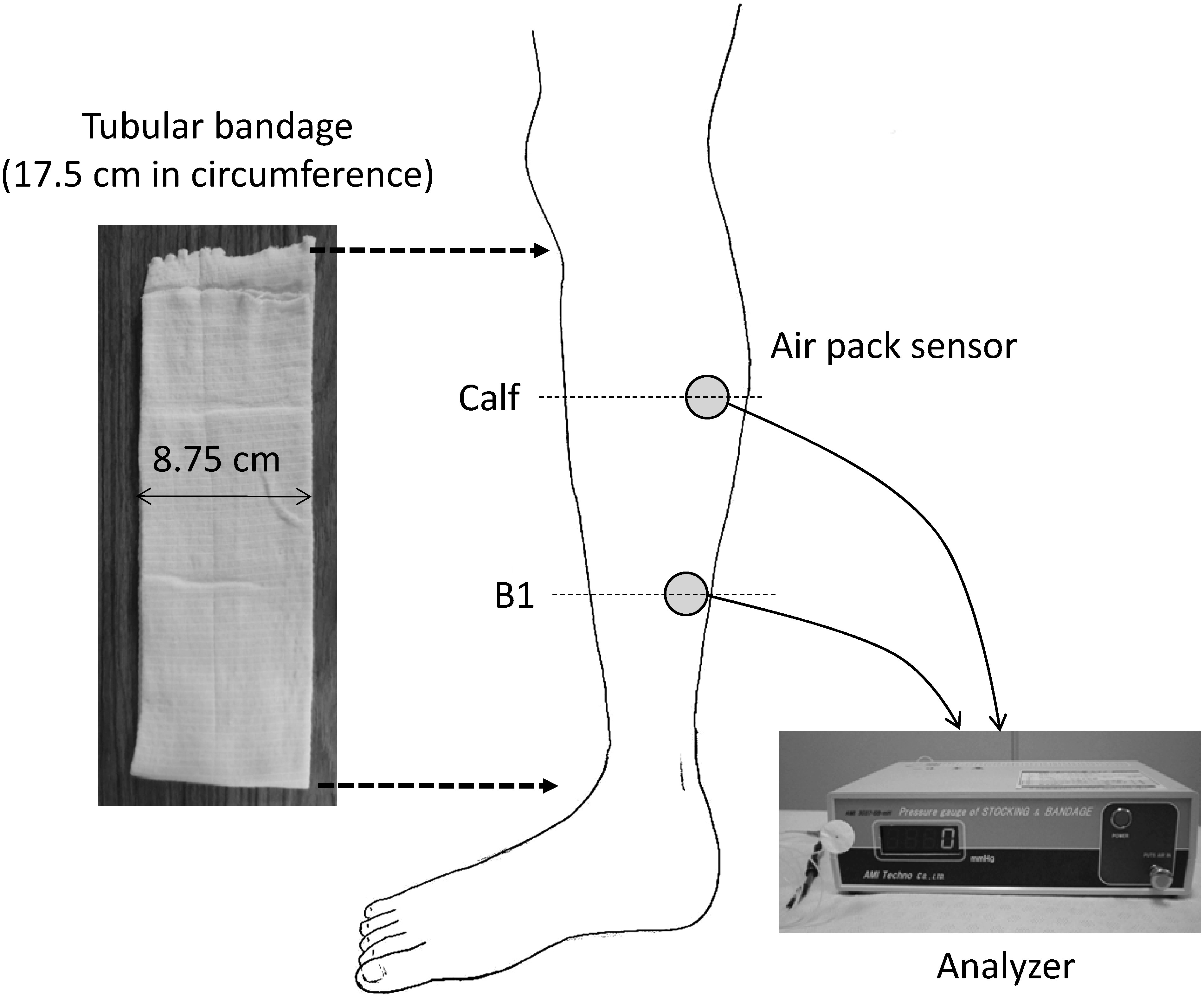
Fig. 1 Points of measurements.

Firstly, two air pack-type sensors were attached to the medial aspect of the right leg, one at the level of the calf at its maximum diameter (C) and another at the level of the transition of the medial gastrocnemius muscle into the Achilles tendon (B1) in each patient (**Fig. 1**). Because the sensor could not be attached properly around the ankle, IP at this level was not measured. The patient first put on the TEB at the level of the fibular head to ankle (single layer). With the patient in a supine position, IPs at the C and B1 levels were measured followed by performing the same measurements with the patient in a standing position. Next, the patients were asked to put on another TEB over the first one (double layer), and IPs were measured as above. For the measurement of IPs, an analyzer (Model AMI-3037-SB, AMI Co., Tokyo, Japan) was used.

### Statistical analysis

Results are expressed as the median (range) or count, unless otherwise indicated. In order to classify the leg size fit for ATS, we used the brochure provided by the manufacturer (AT stocking®, NIPPON SIGMAX Co., Ltd.). The Mann–Whitney U-test was used to test differences in IPs obtained in different positions. The Wilcoxon signed rank sum test was used to test differences in IPs between single- and double-layer TEBs. The correlations between IPs and circumferences were tested using a linear regression analysis. Statistical analyses were performed using JMP 11.0 (SAS Institute, Cary, NC, USA). A p-value <0.05 was considered significant.

## Results

**Figure 2:**
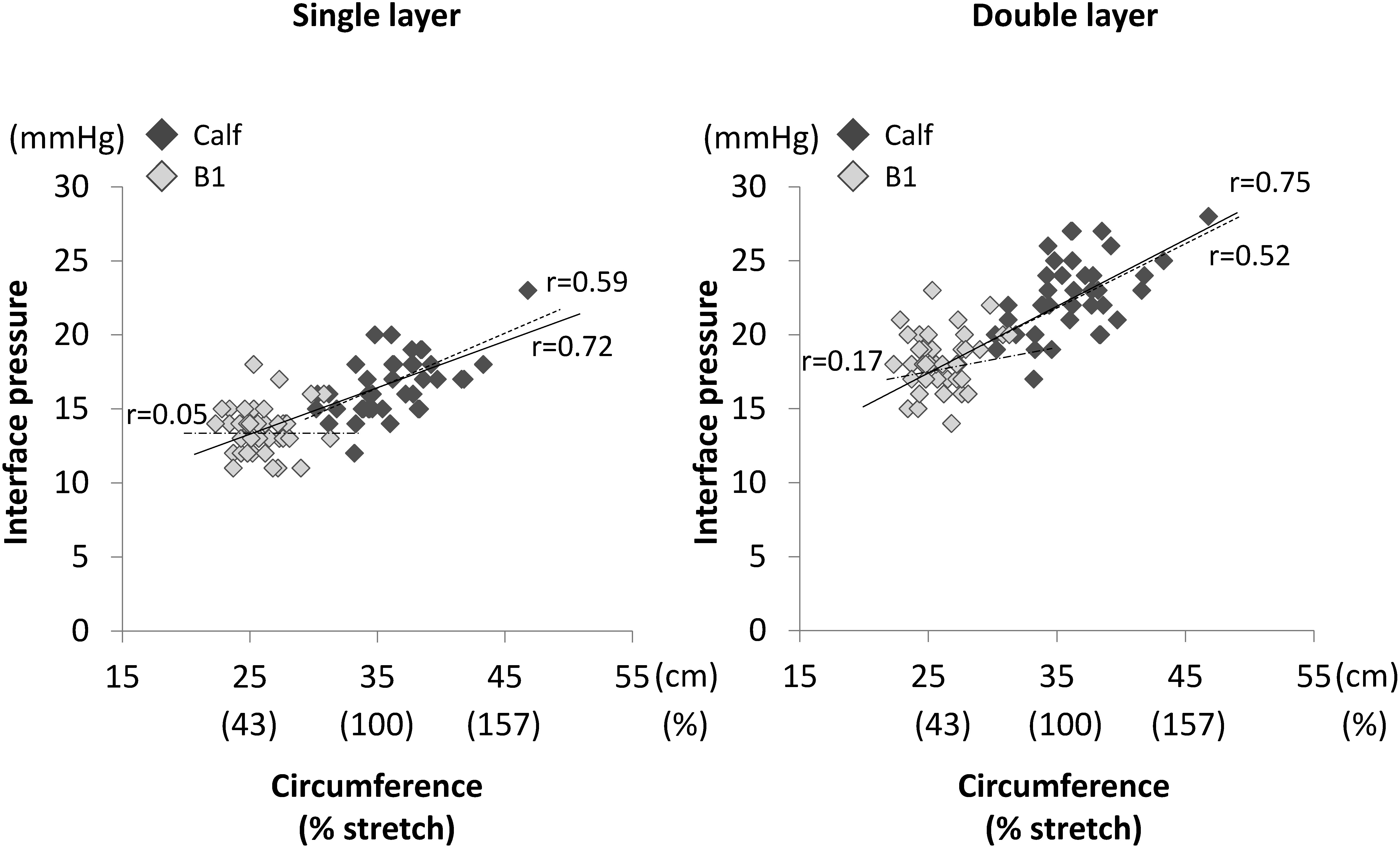
Fig. 2 The correlation between interface pressures and leg circumferences.

The correlations between leg circumferences and IPs in a supine position are shown in **Fig. 2**. When the circumferences and IPs, including both C and B1 levels, were correlated, there was a good linear correlation both with single-layer (r=0.72) and double-layer (r=0.75) TEB. However, when C and B1 levels were assessed separately, circumferences and IPs showed similar linear correlation at the C level for both single- (r=0.59) and double-layer (r=0.52) TEBs, while no such correlation was found at the B1 level.

Solid line: correlation including both the C and B1 levels; dotted line: correlation at the C level; dashed line: correlation at the B1 level.

The IPs obtained using TEBs in various settings are listed in **Table 2**. IPs at the C level were higher than those at the B1 level, and IPs obtained in a standing position were higher than those obtained in a supine position when all other conditions were the same. The IP increased by approximately 1.3 times at both the C and B1 level when double-layer TEB was used. The median static stiffness index, which is defined as the difference between IPs at the B1 level in the supine and standing position,^8)^ increased from 5 to 7 mmHg (p<0.01) when a double layer of TEB was used.

**Table 2:** Table 2 IPs obtained in various conditions

	Single layer		Double layer	
	Calf	B1	Calf	B1
IP (mmHg)				
Supine	17 (12–23)	14 (11–18)^†^	23 (17–28)*	18 (14–23)*^†^
Standing	21 (13–29)^§^	18 (11–26)^†§^	28 (23–40)*^§^	24 (19–34)*^†§^
Static stiffness index (mmHg)		5 (0–11)		7 (2–15)*

^§^: p<0.05 vs. supine position, ^†^: p<0.05 vs. calf, *: p<0.05 vs. single layer. IP: interface pressure

Median IPs according to leg size based on the fit of the ATS are demonstrated in **Table 3**. Using single-layer TEB, the median IP at the C level was 15 mmHg for S-size legs, 17 mmHg for M-size legs, and 18 mmHg for L-size legs. Using a double layer of TEB, the median IP at the B1 level was 18 mmHg for the S-size legs, 18 mmHg for M-size legs, and 19 mmHg for L-size legs. Accordingly, for S-size legs, the median IP at the B1 level was 18 mmHg using a double-layer TEB, and the median IP at the C level was 15 mmHg using a single layer of TEB, which was similar to the pressure profile reported by Sigel et al.^6)^ On the other hand, IPs at the C level were higher than 14 mmHg for M- and L-size legs.

**Table 3:** Table 3 IPs according to leg size based on anti-thrombotic stockings

Circumference at the ankle		S (17.5–20.0 cm)	M (20.0–22.5 cm)	L (22.5–25.0 cm)
Single layer	Calf (mmHg)	15 (12–18)	17 (14–20)	18 (15–23)
	B1 (mmHg)	14 (11–15)	14 (11–16)	14 (11–18)
Double layer	Calf (mmHg)	21 (17–24)	23 (19–27)	24 (10–28)
	B1 (mmHg)	18 (15–21)	18 (15–23)	19 (14–22)

IP: interface pressure

## Discussion

The main findings in this study were as follows: 1) there was a significant linear correlation between IPs obtained using TEB and circumferences, 2) the IP increased approximately 1.3 times by doubling the layer of TEB, and 3) a pressure profile mimicking that of ATS might be created using TEBs in legs of a certain size.

As expected, the simple application of either a single or double layer of TEB did not produce a pressure gradient as recommended to increase venous return. Namely, the IP was higher at the C level than that at the B1 level. Interestingly, Bowling et al. reported that the desired pressure gradient for ATS was achieved in only 14% of legs, and a positive pressure gradient from the calf to ankle was observed in approximately 23% of legs,^9)^ where, if the TEB is doubled only below the calf, then a pressure gradient mimicking ATS might be created in S-size legs. Considering the fact that certain prophylactic effects of ATS to prevent venous thromboembolism could be expected in such conditions, and also considering that the National Institute for Health and Care Excellence guideline recommends the use of stockings producing a calf pressure of 14–15 mmHg to prevent venous thromboembolism in in-hospital patients, which requires a pressure of 14–18 mmHg at the ankle according to the British Standard,^4)^ the pressure profile required to prevent venous thromboembolism itself may need to be revised.

In this study, the correlation between the circumference, i.e., the degree of stretching of the TEB, and IP was linear at the C level, while there was no such correlation at the B1 level. In results that might be interpreted as the correlation at each level representing a different phase of hysteresis, this is probably because we used linear regression analysis for each level. Another possible explanation for this result is “floating” of the TEB at the B1 level because the B1 level is recessed between the calf and ankle and because the degree of depression varies widely depending on the leg shape.

### Limitation

Since this study was a single-center study that included a limited number of patients, reaching a definitive conclusion is difficult. The validity of the pressure profile of ATS is generally determined using the IP at the ankle level. However, we could not find an appropriate place to attach the sensor around the ankle in which little flat and non-bony places could be found. This might have prevented the obtaining of conclusive results. Since there are a wide variety of commercially available TEBs with different sizes and made of different materials, the current results may not be generalizable; therefore, the circumference-pressure relationship needs to be clarified for each TEB. Furthermore, because the pressure profile may not the only factor determining anti-thrombotic properties, validity of the use of TEBs instead of ATS should be tested in future clinical trials.

## Conclusion

The IP achieved using TEBs linearly correlated with calf circumference, and using double-layer TEB increased the IP by up to 1.3 times. Using these characteristics, it seems feasible to create a pressure profile mimicking that of ATS using TEBs.
